# A Multilevel Computational Characterization of Endophenotypes in Addiction

**DOI:** 10.1523/ENEURO.0151-18.2018

**Published:** 2018-07-17

**Authors:** Vincenzo G. Fiore, Dimitri Ognibene, Bryon Adinoff, Xiaosi Gu

**Affiliations:** 1 School of Behavioral and Brain Sciences, University of Texas at Dallas, Richardson, TX 75080; 2 Department of Computer Science and Electronic Engineering, University of Essex, Colchester CO4 3SQ, United Kingdom; 3 Department of Information and Communication Technologies, Universitat Pompeu Fabra, Barcelona 08018, Spain; 4 University of Texas Southwestern Medical Center, Dallas, TX 75390; 5 VA North Texas Health Care System, Dallas, TX 75216; 6 Department of Psychiatry, Icahn School of Medicine at Mount Sinai, New York, NY 10029

**Keywords:** addiction, neural model, phenotyping, reinforcement learning

## Abstract

Addiction is characterized by a profound intersubject (phenotypic) variability in the expression of addictive symptomatology and propensity to relapse following treatment. However, laboratory investigations have primarily focused on common neural substrates in addiction and have not yet been able to identify mechanisms that can account for the multifaceted phenotypic behaviors reported in the literature. To fill this knowledge gap theoretically, here we simulated phenotypic variations in addiction symptomology and responses to putative treatments, using both a neural model, based on cortico-striatal circuit dynamics, and an algorithmic model of reinforcement learning (RL). These simulations rely on the widely accepted assumption that both the ventral, model-based, goal-directed system and the dorsal, model-free, habitual system are vulnerable to extra-physiologic dopamine reinforcements triggered by addictive rewards. We found that endophenotypic differences in the balance between the two circuit or control systems resulted in an inverted-U shape in optimal choice behavior. Specifically, greater unbalance led to a higher likelihood of developing addiction and more severe drug-taking behaviors. Furthermore, endophenotypes with opposite asymmetrical biases among cortico-striatal circuits expressed similar addiction behaviors, but responded differently to simulated treatments, suggesting personalized treatment development could rely on endophenotypic rather than phenotypic differentiations. We propose our simulated results, confirmed across neural and algorithmic levels of analysis, inform on a fundamental and, to date, neglected quantitative method to characterize clinical heterogeneity in addiction.

## Significance Statement

Addiction is known to encompass heterogeneity in its development, maintenance, and treatment response. While previous work has mostly focused on the common mechanisms underlying vulnerabilities in addiction at a group level, the neurocomputational causes for such intersubject variability in addition are not well understood. To fill this knowledge gap, we combine a neural and a reinforcement learning (RL) model to reveal that the balance between neural circuits or computational control modalities characterizes the presence of behavioral phenotypes in addiction. The presence of converging effects, validated across neural and algorithmic levels of analysis, informs on a quantitative method to characterize clinical heterogeneity and potentially helps future development of precision treatments.

## Introduction

Addiction is known to encompass a wide range of individual behavioral differences (i.e., phenotypes) in development, maintenance and severity of symptoms, and treatment response (Everitt and Robbins, 2016). Previous investigations into the mechanisms underlying this heterogeneity of behaviors have identified two fundamental neurocomputational alterations correlated with vulnerability in the development and severity of addictive behaviors (Garrison and Potenza, 2014; Jupp and Dalley, 2014; Belin et al., 2016). These neural and computational intersubject differentiations (i.e., endophenotypes) include (1) a dysregulation of D2 receptors in the striatum (Morgan et al., 2002; Nader and Czoty, 2005; Dalley et al., 2007; Flagel et al., 2014) and (2) an alteration of learning rates within a reinforcement-learning framework (Gutkin et al., 2006; Piray et al., 2010). However, these endophenotypic differences are found across a wide spectrum of dissociable phenotypes, so that the same neural or computational mechanism is used to account for separable behavioral traits. For instance, different forms of striatal D2 dysregulation are found in individuals differing in terms of their impulsivity (Dalley et al., 2007; Volkow et al., 2007), social dominance (Morgan et al., 2002; Gould et al., 2014), motor reactivity or preference for novelty (Flagel et al., 2010, 2014), or sensitivity to rewards (Belcher et al., 2014). Each of these behavioral traits is separately correlated with development of addiction, but they do not necessarily coexist in the same individuals (cf. novelty seeking and impulsivity: Ersche et al., 2010; Molander et al., 2011; Belin and Deroche-Gamonet, 2012). This mismatch between few known endophenotypic differences and a wide variety of multifaceted, dissociable, behavioral phenotypes suggests there are yet unknown neural and computational mechanisms that are responsible, alone or in interaction, for the reported behavioral differentiations. Finally, investigations into intersubject variability often emphasize the initial stage of addiction development (but see Belin et al., 2008; Economidou et al., 2009; Pelloux et al., 2015). Yet, individual differences also exist in treatment response, resulting in diverse relapse patterns among individuals showing similar severity of symptoms. These differences have not been so far addressed in previous neural or computational models.

Here, we propose a theoretical investigation into the interaction between ventral and dorsal cortico-striatal circuits and the associated behavioral control modalities. Several studies have emphasized that addiction is associated with alterations of ventral and dorsal cortico-striatal circuits, and of motivations and habits (Volkow and Morales, 2015; Everitt and Robbins, 2016; Koob and Volkow, 2016). However, the role played by the interaction between the two neural circuits or between the two behavioral control modalities in generating intersubject variability in addiction, has been so far neglected. To investigate this interaction, we use two models to simulate neural dynamics and algorithmic (or normative) choice selections in a multiple-choice task involving drug and non-drug rewards. Then we test these models under different conditions of circuit or control modality dominance (i.e., simulated endophenotypes). Consistently with previous models, we assume addictive substances hijack the healthy reward prediction error signal (Schultz et al., 1997) by triggering extra-physiologic dopamine bursts (Nestler and Aghajanian, 1997; Koob and Volkow, 2016). These dopamine activities signal the presence of an aberrant unexpected reward, leading to the repetition of drug-related actions and escalation of consumption (Redish et al., 2008; Dayan, 2009). In our neural model, this process of reinforcement learning (RL; Sutton and Barto, 1998) is mediated by extra-physiologic changes in cortico-striatal connectivity weights (Hyman et al., 2006; Haber, 2008; Koob and Volkow, 2016). These changes in turn aberrantly affect circuit gain and the stability of both ventral and dorsal cortico-striatal circuits, disrupting their respective roles in encoding and selecting goal-directed behaviors (Balleine, 2005; Balleine and O'Doherty, 2010; Gruber and McDonald, 2012) and habitual responses (Yin et al., 2004; Balleine and O'Doherty, 2010). A similar effect is assumed for our algorithmic model, where overevaluation of drugs and related RL affect the two control modalities, termed model-based and model-free, that approximate ventral/goal-oriented and dorsal/habitual implementations (Dolan and Dayan, 2013; Voon et al., 2017). As a result, and consistently with previous formulations of RL models of addiction (Redish et al., 2008; Piray et al., 2010; Gillan et al., 2016), both the planned evaluation of known action-outcome contingencies, represented in an internal model of the world, and the reactive immediate motor responses are biased toward drug-related selections.

Based on these assumptions, our models show that phenotypic differentiation in addiction development and treatment response can emerge as a function of the interaction between ventral and dorsal circuits or model-based and model-free control modalities. Our simulated results offer a proof-of-concept that this interaction is a candidate independent neural and computational mechanism underlying addiction vulnerability, putatively characterizing three different endophenotypes differing in the likelihood to develop addiction, severity of symptoms and treatment response. We suggest this neurocomputational mechanism could interact with both previously described D2 receptors dysregulation in the striatum (Dalley et al., 2007; Flagel et al., 2014) and altered learning rates (Gutkin et al., 2006; Piray et al., 2010) to generate the variety of dissociable behavioral traits reported in literature as associated with addiction vulnerabilities.

## Materials and Methods

In brief, we present two complementary models simulating endophenotypic differences and their effects on addiction development and treatment response. In the models, intersubject differences are expressed in terms of either neural circuit dominance (i.e., ventral or dorsal circuit) or control modality dominance (i.e., model-based or model-free) in determining behavioral selections. The resulting phenotypes are tested in environments granting free access to a simulated substance of addiction, as usually implemented in laboratory studies. In particular, we compare our simulated phenotypic variability with the results described in a recent study investigating individual differences in rats self-administrating the stimulants cocaine or a designer drug, a dopamine- and mixed dopamine-norepinephrine reuptake inhibitor, respectively (Gannon et al., 2017). We selected this study because it highlights how different drugs, dosages, and tasks result in different ranges of phenotypic differentiation. For instance, an initial acquisition phase, over a 10-d period, shows compulsive behavior developed in up to 75% rats self-administering cocaine and 87.5% of those exposed to the designer drug. Furthermore, under a condition of fixed ratio (=5) schedule, the study shows self-administration varied significantly among subjects. A subset of rat population, termed high responders, self-administered cocaine up to 60% more times in comparison with a different subset, termed low responders, depending on dosage (cf. Gannon et al., 2017, and their Fig. 3). Importantly, the task setup chosen for both of our proposed models involves the selection of a drug reward over explicit non-drug-related alternatives; in contrast, the chosen empirical study utilizes a time-out responding paradigm, where the only explicit non-drug-related behavior (a lever-press) is not rewarded. As for most studies simulating addiction (Redish, 2004), we believe the choice to present our simulated agents with a richer set of options (i.e., more than one) does not invalidate a parallel between simulated and real data. We consider the simulated competing options as a proxy for the many conflicting stimuli and associated behaviors that animals have access to, even in the limited environment of a standard operant conditioning chamber. Thus, our focus is on perturbing the balance between the dorsal/model-free and the ventral/model-based systems, to compare our simulated behavioral differentiations in the escalation and compulsive selection of drug-related actions with the data reported in the chosen laboratory study.

The two models comprise a neural mass model that has been validated and described in the context of choice behavior and dopaminergic modulation (Fiore et al., 2016, 2018; Hauser et al., 2016) and a normative or algorithmic model based on standard RL schemes (Sutton and Barto, 1998). In the neural model, addiction and treatment response are modeled through DA-dependent associative plasticity in both ventral and dorsal circuits. In the RL model, aberrant learning is modeled using a duplex of model-based and model-free schemes that competed for control over action selection. The model-based scheme entails learning a model of the environment (in the form of probability transition matrices among states) that is used to compute value functions under the Bellman optimality principle (Bellman, 1966). The equivalent model-free scheme uses prediction error-based learning to directly acquire the value of state action pairs. Both neural and RL models are tested under four successive stages or phases: (1) before exposure to the simulated drug (termed pre-drug); (2) learning of addictive behavior (termed addiction); (3) simulated ideal therapeutic interventions (termed treatment) that partially revert the learning of the previous phase; and finally, (4) reinstated access to the simulated drug following each treatment (termed relapse). The simulated treatments are conceived to emphasize endophenotypic response and relapse differentiation; and therefore, they predominantly affect only one control system, targeting either the goal-oriented/model-based or the habitual/model-free. The former treatment is assumed to modify only the internal model of the environment and related selection of action-outcome contingencies performed in the ventral circuit. The latter treatment represents a condition in which the model of the world of the agent remains mainly unaltered, but the acquired drug-related stimulus-response associations are disrupted, thus preventing the agent from exhibiting habitual responses (cf. Doll et al., 2009).

The unique aspect of this complementary modeling approach is that converging results from neural and algorithmic models can validate each other, as process and implementation theories (i.e., synaptic and dynamical mechanisms) complement the normative principles formalized in the RL model.

### Neural field model

#### Basic model architecture and parameterization

**Figure 1.**
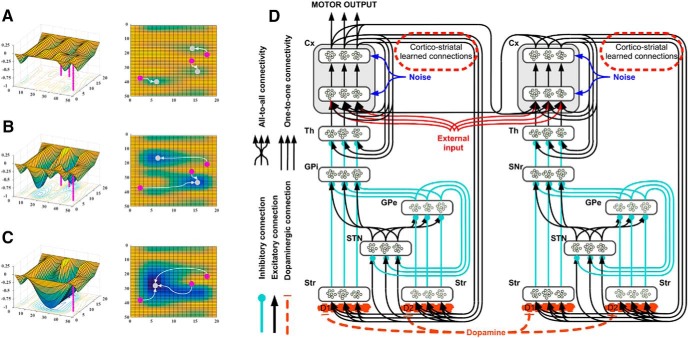
Illustrative representation of energy landscapes and neural architecture of the model. ***A–C***, These representations of energy landscapes are meant to illustrate differences in the temporal responses provided by neural systems. Depending on the energy landscape, three arbitrary inputs (magenta dots) are transformed into different stable states (gray dots). Learning processes increase or decrease the strength of the connections among nodes in a network, thereby altering its energy landscape and reshaping temporal responses toward existing attractors. Attractors are defined as low-energy states (bottom of the basins) at the end point of the temporal responses to multiple starting inputs. ***A***, The landscape is characterized by multiple shallow attractors: these allow slow temporal responses, transforming multiple inputs into multiple weakly stable states. Noise and changes in the incoming input easily determine new responses toward different attractors. ***B***, In this second illustrative configuration, steep and vast attractors characterize the energy landscape, allowing quick state transitions toward two equilibrium points. This new configuration is able to resist noise and minor changes in the incoming input, and, at the same time, allows a differentiation of inputs in two broad categories. ***C***, Finally, the third energy landscape illustrates the presence of a parasitic attractor, exemplifying the condition of addiction: all inputs fall now at the bottom of a single steep basin. Under this condition, noise and changes in the incoming input determine temporal responses that keep falling in the same attractor, therefore preventing the system from executing different behaviors. ***D***, Neural architecture used to simulate neural dynamics and behavior for the mean field neural model. The activity in the dorsal cortico-striatal circuit is responsible for the motor output of the system (left circuit), while activity in the ventral cortico-striatal circuit is responsible for goal selections (right circuit). The two systems bias each other via corticocortical connectivity and learning processes affect the weights of the connections between the two cortical outputs and the striatum in their corresponding circuits. The components in the architecture are labeled as follows: cortex (Cx), thalamus (Th), globus pallidus pars externa and interna (GPe and GPi), substantia nigra pars reticulata (SNr), subthalamic nucleus (STN), and striatum (Str), divided into two areas enriched by either D1 or D2 dopamine receptors.

In cortico-striatal circuits, the signal processed in the cortex is conveyed toward its respective area of the striatum, processed in basal ganglia and finally relayed to the same cortical area where it originated, via thalamus (Haber, 2003; Draganski et al., 2008; Jahanshahi et al., 2015). Thus, despite diverging in terms of the information processed, e.g., sensorimotor or rewards and outcomes, these circuits are characterized by similar computational dynamics (Obeso et al., 2014). Temporal responses in recurrent neural networks co-occur with state transitions or input transformations that are often described in terms of energy landscapes (Fig. 1*A–C*). If multiple inputs or initial states generate transitions toward the same final state, this is termed attractor state (Amit, 1989). In recurrent networks such as cortico-striatal circuits, learning processes modulate the circuit gain, thereby affecting the strength of the attractor states and the overall stability of the system (Fiore et al., 2015, 2016; Hauser et al., 2016).

We simulate the temporal responses in cortico-striatal circuits in a neural model (for illustrative representation of the neural architecture, see Fig. 1*D*). This neural model simulates mean-field activity (Deco et al., 2008) within multiple channels of both dorsal and ventral cortico-striatal loops. A continuous-time differential equation simulates changes over time $\left(\tau_{g}\right)$ of the average action potential ${(u}_{j})$ of a pool of neurons (Eq. 1), and a positive transfer function (Eq. 2) converts this action potential in the final activation of the pool ($y_{j}$). Finally, the plasticity of the connections ($w_{ij}$) between cortex and striatum is characterized by DA-dependent Hebbian learning, corrected with a constant threshold (*th*) as defined in Equation 3. The resulting rule strengthens the connections among all active nodes in the cortex and those active in the striatum and weakens the connections among nodes showing opposite activation status.

(1) $$\tau_{g}{\dot{u}}_{j}=-u_{j}+b_{j}+\left(\epsilon +\lambda d\right)\sum w_{ji}y_{i}$$

(2) $$y_{j}={\left[\tanh\left(u_{j}-\theta \right)\right]}^{+}$$

(3) $${\Delta w}_{ij}=\eta \left({\left[y_{i}-th\right]}^{+}{\left[y_{j}-th\right]}^{+}{\left[d-th\right]}^{+}\right)-\zeta \left({\left[th-y_{i}\right]}^{+}{\left[th-y_{j}\right]}^{+}{\left[d-th\right]}^{+}\right)$$

The input ($\sum w_{ji}y_{i}$), reaching each node in the neural network is modulated by two coefficients λ and ϵ. These regulate the ratio between the signal affected by the presence of dopamine release d and the amount of signal that is computed independent of dopamine release. For most units, the values of the two coefficients are set to λ=0 and ϵ=1, with the exception of the simulated striatal units, where these parameters are set to $\left[\lambda =1.4,\epsilon =0.2\right]$and [λ=-0.5,ϵ=0.6], to simulate the differential effect dopamine has, depending on the most prevalent receptor type ( > 1 and λ < 0 for D1 and D2 receptors, respectively). Due to the different effects the dopamine receptors have on the activity of the simulated neurons, the drug-induced dopamine-dependent Hebbian learning significantly affects D1-enriched units in the striatum, while having negligible effects on D2-enriched units (Gerfen and Surmeier, 2011; Volkow and Morales, 2015).

#### Simulating different addiction phenotypes and treatment effects

Agents controlled by the neural model are immersed in a simplified environment and can select among three arbitrary actions or inactivity (cf. nonstationary three armed bandit environment). The selection of the actions is conducted in the circuit simulating the dorsal cortico-striatal activity, and it is considered completed if the neural activity of any of the units in the external layer of the simulated cortex (Fig. 1*D*) is maintained for at least 2 s. Ventral and dorsal circuits interact, both ways, via corticocortical connectivity. Therefore, the activity in the simulated ventral circuit biases action selection in the dorsal circuit and the selection of actions in the dorsal circuit biases the activity in the ventral circuit. To test our hypothesis about the effect these reciprocal biases have on choice behavior, we assumed corticocortical weights do not vary over time and we tested eleven combinations for the parameters determining their weights, as $w_{ji}$= [0.02–0.2], [0.03–0.17], [0.03–0.15], [0.05–0.15], [0.07–0.13], or [0.1–0.1] (and symmetrical). This spectrum of weights describes the strength of the biases between the two major circuits, thereby characterizing either a balanced condition or a dominance of one of the two circuits. We report the effects in terms of behavioral responses for these putative endophenotypes and test each of these with thirty noise seeds, random inputs and under four stages, to allow within phenotype comparisons. The first stage, “pre-drug,” represents an assessment of behavior before any drug or reward is introduced, as the three available inputs randomly change their value to determine a nonstationary order of preferences. Under the second stage, termed “addiction,” one action is associated with the administration of a simulated addictive substance, triggering DA phasic responses and associated Hebbian learning in cortico-striatal connections of both ventral and dorsal circuits. For the third stage, termed “treatment,” we simulate the effects of deprivation coupled with one of two hypothetical treatments targeting either the dorsal or the ventral cortico-striatal circuits. The treatments are simulated by reverting the learning process in either the dorsal or the ventral cortico-striatal circuit, respectively, representing an intervention that would block or extinguish either the habitual drug-related response (an ideal behavioral treatment) or the drug-related emotional and value association (an ideal cognitive treatment). The dorsal treatment brings back the pre-drug configuration in the dorsal circuit and keeps the configuration reached under the addiction stage for the ventral circuit. The ventral treatment is achieved with the opposite intervention. Finally, during the fourth stage, termed “relapse,” we reintroduce access to the simulated addictive substance, inducing relapse. For this stage, relapse time is defined as the time required to reinstate the configuration of cortico-striatal weights found at the end of the addiction stage.

### RL model

#### Basic model architecture and parameterization

In this model, we assume that the behavior of the agent relies on a hybrid model (Daw et al., 2011) that learns and computes the value of choices (actions, $a_{t}$) under each condition (state, s_t_). Value is defined as a quantity that combines short and long-term expected rewards and negative outcomes when a specific strategy of action is followed (policy, π). It is formally defined as:

(4) $$Q^{\pi }\left(s_{t}a_{t}\right)=r\left(s_{t}a_{t}\right)+E\left[\sum_{i}^{\infty }\gamma^{i}r\left(s_{t+i},a_{t+i}=\pi \left(s_{t+i}\right)\right)|s_{t},a_{t}\right]$$

In Equation 4, $r\left(s,a\right)$ denotes the instantaneous reward received when action *a* is performed in state *s*. *γ* is a discount factor, comprised between 0 and 1, which defines the trade-off between immediate and long-term rewards. The value of a state given the policy is defined as $V^{\pi }(s)=\underset{a}{\max}Q^{\pi }\left(s,a\right)$. For each environment, there is an optimal policy $\pi^{*}\left(s\right)$, which maximizes the value $V^{\pi^{*}}(s)$ for every state (Sutton and Barto, 1998).

The environment can be completely characterized through the state transitions distributions $p\left(s_{t+1}=s|s_{t},a_{t}\right)$, and the expected rewards $E\left(r|s,a\right)=R\left(s,a\right)$. These two functions together represent a model of the environment. Model-based behaviors compute $Q^{\pi }\left(s_{t}a_{t}\right)$ and the policy relying on such functions, at each state, following the Bellman equation (Daw and Dayan, 2014):

(5) $${\text{Q}}^{{\text{π}}^{\text{∗}}}\left({\text{s}}_{\text{t}}\text{,}{\text{a}}_{\text{t}}\right)\text{=R}\left({\text{s}}_{\text{t}}\text{,}{\text{a}}_{\text{t}}\right)\text{+γ}\sum_{\;\text{s}}\left[\text{p}\left({\text{s}}_{\text{t+1}}\text{=s|}{\text{s}}_{\text{t}}\text{,}{\text{a}}_{\text{t}}\right)\underset{a}{\max}{\text{Q}}^{{\text{π}}^{\text{∗}}}\left(\text{s,a}\right)\right]$$

The model-based component learns the transition distributions and the expected rewards during the interaction with the environment. Thus, differently from other hybrid models (Daw et al., 2005; Keramati et al., 2011; Pezzulo et al., 2013), the quality of *Q* value estimation at any given moment depends on the experience the agent acquired up to that point in time. To compute value estimation ($Q_{MB}$), this bounded (Gershman et al., 2015) component applies at each step the Bellman equation (Eq. 5) a limited number of times $\left(N_{PS}=50\right)$ to states sampled stochastically following a heuristic for efficient state update selection. The algorithm is an early-interrupted variation of the Prioritized Sweeping algorithm (Moore and Atkeson, 1993) with stochastic state update selection. Crucially, our model-based component does not accumulate the variations of *Q* values over time, and restarts the computation after each step (desJardins et al., 1999). This choice is meant to instate a plausible bounded rationality for our model which can account for the cognitive costs and ensuing limits of integrating old and new information about the environment, while updating and extending a complex plan to navigate it. This implementation is suitable for a bounded rational model-based component that shows controlled stochasticity of deliberation performances in nontrivial environments. This choice allows to test the effects of the hypothesized endophenotypic differentiation in an environment characterized by higher degree of complexity in comparison with both the one chosen for the neural model and those described in the literature of RL models of addiction. In particular, we consider drug consumption to be associated with complex after-effects that make it difficult to predict the overall result of pursuing the related action course.

In comparison with other hybrid models such as Dyna and Dyna2 (Sutton, 1990; Silver et al., 2016), the proposed architecture does not share *Q* values between model-based and model-free components, nor it requires that the two processes share the same state representations. The two components separately represent their *Q* values and integrate them in a later phase. This decoupling is assumed to result in a more biologically plausible agent (Daw and Dayan 2014), and it is essential for the simulations of two separate treatments, essential requirement to establish a comparison with the behavior simulated with the neural model. In contrast with previous work using a hybrid Dyna-like architecture and prioritized sweeping algorithm, where the sharing of the *Q* values explained the appearance of model based drug oriented behavior (Simon and Daw, 2012), in our simulations this model based addiction emerges in independent model-free and model based components. Thus, addiction behavior results from the joint effect of high reward (i.e., the drug), a limited number of stochastically selected policy updates and limited knowledge of the environment.

The model-free component has been implemented using the Q-Learning algorithm in tabular form (Watkins and Dayan, 1992). Q-learning updates initial state value estimations as follows:

(6) $$Q_{MF}{\left(s_{t},a_{t}\right)}_{new}=Q_{MF}{\left(s_{t},a_{t}\right)}_{old}+\alpha \delta_{t}$$

(7) $$\delta_{t}=R\left(s_{t},a_{t}\right)+\gamma \,\max_{a\prime }\left[Q_{MF}\left(s_{t+1},a\prime \right)\right]-Q_{MF}\left(s_{t},a_{t}\right)$$

where α is a learning factor comprised between 0 and 1. Our hybrid model computes choice values in a fashion that balances model-free (MF in the equations) and model-based (MB in the equations) components depending on a parameter β. Six values (1, 0.8, 0.6, 0.4, 0.2, 0) are used for this parameter to simulate different endophenotypes, on a spectrum between purely model-based (β= 1) and purely model-free (β = 0) RL.

To allow exploration, the action to execute is selected randomly 10% of the times. This exploration factor is kept constant to support adaptation to a changing environment (Singh et al., 2000) and to simulate the continuous update of knowledge necessary to cope with ecological environments. The remaining 90% of the times, actions are determined by maximizing Q_MX_(s,a) in a strategy defined as ε-greedy (ε = 0.1). These values are produced by combining the values computed by the model-based and model-free components:

(8) $$Q_{MX}\left(s,a\right)=\beta Q_{MB}\left(s,a\right)+(1-\beta )Q_{MF}\left(s,a\right)$$

The choice for a fixed balance between model-based and model-free requires minimal assumptions on their interaction and has been used in recent RL architectures (Silver et al., 2016).

#### Simulating different addiction phenotypes and treatment effects

**Figure 2.**
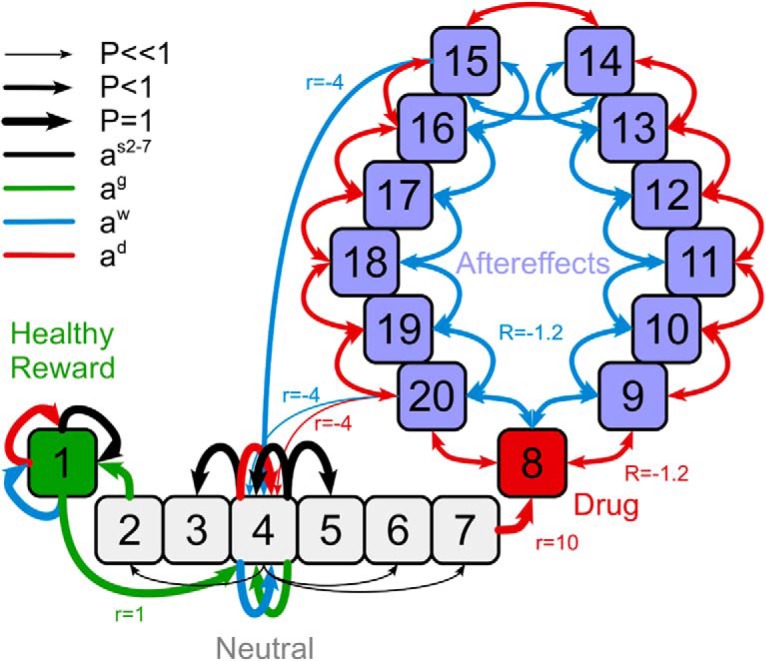
Illustrative representation of the environment used for the RL model of addiction. The states are disposed in a linear arrangement: on one extreme is a healthy reward state (1), on the opposite side a drug state (8) followed by twelve aftereffects states (9–22). Healthy reward and drug states are separated by six neutral states (2–7). The agent can traverse between nearby neutral states. From the two borders of the central segment of neutral states, an agent can enter the healthy reward state (from state 2), securing a moderate reward (Rg = 1), or the drug state (from state 7), receiving an initial high reward (Rd = 10, during the phase of addiction) and a series of sparse but temporally extended negative outcomes, characterizing the aftereffects states. The presence of negative outcomes makes entering the drug and aftereffects area suboptimal during all experimental phases (see optimal policy in Table 3). From both the goal state and the drug/aftereffects segment the agent is then returned to the middle of the neutral segment. In this representation, we explicitly portray the transitions related to states 1 (healthy reward), 4 (neutral), and 15 and 20 (drug aftereffects) for illustrative purposes. Line width represents related transition probability value. Line and text color represent the action class (a_s_, a_g_, a_w_, a_d_). Neutral states are navigable with actions a_s2-7_, which are deterministic for adjacent state while have high chance of failing for distant states. From the neutral states the agent can reach: (1) the healthy reward, if executing action a_g_ when in state 2; and (2) the drug state (8) and aftereffects area (state 9–22), if executing action a_d_, when in state 7. From the healthy reward area, the agent can issue again a_g_, receiving a reward of 1 and going back to the center of the neutral area, state 4. By entering the drug area, the agent receives a reward of 10. Action results in the drug/aftereffect area are probabilistic: the agent can reach a nearby state in the area or leave the area and reach the center of the neutral state. Leaving the drug/aftereffects area has a cost of -4, whereas every other transition inside the area costs -1.2. For a full description of transitions and their probability distribution in the environment, see Tables 1, 2, 4, 5).

In comparison with the simulations characterizing the neural model, a more complex environment is in use for the RL model to highlight how our endophenotypic differentiations can also affect the likelihood to develop addiction. This environment is characterized by a total of 20 states divided into four different types (Fig. 2): (1) healthy rewards (i.e., normal rewards that are not directly associated with drugs); (2) neutral states (no reward or negative outcome); (3) drug-related states, which give a high reward but are followed by multiple (4) drug aftereffects, characterized by small negative outcomes. Similar to the neural model investigations, the agent deals with environment variations meant to simulate four phases of addiction: initial pre-drug phase (f1); addiction (i.e., the drug becomes accessible for the first time, f2); treatment (f3); relapse (i.e., second drug exposures; f4). Under the initial pre-drug phase (d_init_ = 50 steps), the agent does not receive any reward or negative outcome by entering the drug-related and aftereffects area, but a moderate reward is assigned (R_g_ = 1) by accessing the healthy reward state. Under the phases of addiction and post-treatment addiction (d_tpy_ = 1000 steps), the agent can also receive a high reward, after accessing a drug-related state (R_d_ = 10). The drug state always leads to a series of randomized state transitions among the aftereffects states (R_a_ = -1.2) and simulates generic negative consequences associated with addiction. The agent can occasionally leave this aftereffect area of the environment (Fig. 2) to reach a neutral state, at the price of a further negative outcome (R_a_ = -4). Under the treatment phase (d_tpy_ = 1000 steps), the drug-related state results in a negative outcome (R_dt_ = -1; Tables 1, 2, column f3), thus increasing the chances the agent stops pursuing this state. To allow for a comparison with the results in the neural model, we simulate a model-based and model-free treatment by manipulating the learning factor of the nontreated control modality, decreasing it: α_Ctpy_ = 0.01 * α. Under the relapse phase, we measure the simulated time required by the agents to reach at least 95% of drug-related action preference as recorded under the addiction phase, after the drug is introduced again in the environment. This threshold is used to measure the percentage of agents relapsing, as well as the time required to complete the relapse, per endophenotype.

**Table 1.** Environment transition probabilities across endophenotypes controlled by the RL model

Transition Description	Probability for each phase				
	P (f1)	P (f2)	P (f3)	P (f4)	
P(s=i|s=i,a=a^s=i^), i neutral state	1	1	1	1	From Neutral States
P(s=i+j|s=i,a=a^s=i+j^),j=+1/-1, i neutral state, i+j neutral state	0.99	0.99	0.99	0.99	
P(s=i|s=i,a=a^s=i+j^) ,j=+1/-1, i neutral state, i+j neutral state	0.01	0.01	0.01	0.01	
P(s=i+k|s=i,a=a^s=i+k^),k!=+1/-1, i neutral state, i+k neutral state	0.0001	0.0001	0.0001	0.0001	
P(s=i|s=i,a=a^s=i+k^),k!=+1/-1, i neutral state, i+k neutral state	0.9999	0.9999	0.9999	0.9999	
P(s=i|s=i,a=a^w^), i neutral state	1	1	1	1	
P(s=1|s=2,a=a^g^)	1	1	1	1	
P(s=i|s=i,a=a^g^), i!=2 neutral state	1	1	1	1	
P(s=8|s=7,a=a^d^)	1	1	1	1	
P(s=i|s=i,a=a^d^), i!=7 neutral state	1	1	1	1	
P(s=i|s=i,a=a^g^), i drug/aft state	0.999	0.999	*0.8*	0.999	From Drug/aft States
P(s=4|s=i,a=a^g^), i drug/aft state	0.001	0.001	*0.2*	0.001	
P(s=i|s=i,a=a^s=*^), i drug/aft state	0.999	0.999	*0.8*	0.999	
P(s=4|s=i,a=as=*), i drug/aft state	0.001	0.001	*0.2*	0.001	
P(s=j|s=i,a=a^w^), i!=15 drug/aft state, j next or previous drug/aft state	0.4995	0.4995	*0.4*	4.995	
P(s=4|s=i,a=a^w^), i!=15 drug/aft state	0.001	0.001	*0.2*	0.001	
P(s=14/16|s=15,a=a^w^)	0.2	0.2	*0.15*	0.2	
P(s=4|s=15,a=a^w^)	0.6	0.6	*0.7*	0.6	
P(s=j|s=i,a=a^d^), i drug/aft state, j next drug/aft state	0.745	0.745	*0.6*	0.745	
P(s=j|s=i,a=a^d^), i drug/aft state, j previous drug/aft state	0.245	0.245	*0.2*	0.245	
P(s=4|s=i,a=a^d^), i drug/aft state	0.01	0.01	*0.2*	0.01	
P(s=4|s=1,a=a^g^)	1	1	1	1	Goal
P(s=1|s=1,a=a^s=*^)	1	1	1	1	
P(s=1|s=1,a=a^w^)	1	1	1	1	
P(s=1|s=1,a=a^d^)	1	1	1	1	

Changes during phases in italic.

**Table 2.** Environment rewards across endophenotypes controlled by the RL model

Transition Description	Probability for each phase				
	P (f1)	P (f2)	P (f3)	P (f4)	
T(s=i|s=i,a=a^s=i^), i neutral state	0	0	0	0	From States
T(s=i+j|s=i,a=a^s=i+j^), j=+1/-1, i neutral state, i+j neutral state	0	0	0	0	
T(s=i|s=i,a=a^s=i+j^), j=+1/-1, i neutral state, i+j neutral state	0	0	0	0	
T(s=i+k|s=i,a=a^s=i+k^),k!=+1/-1, i neutral state, i+k neutral state	-0.3	-0.3	-0.3	-0.3	
T(s=i|s=i,a=a^s=i+k^),k!=+1/-1, i neutral state, i+k neutral state	0	0	0	0	
T(s=i|s=i,a=a^w^), i neutral state	0	0	0	0	
T(s=1|s=2,a=a^g^)	0	0	0	0	
T(s=i|s=i,a=a^g^), i!=2 neutral state	0	0	0	0	
T(s=8|s=7,a=a^d^)	0	*10*	*-1*	*10*	
T(s=i|s=i,a=a^d^), i!=7 neutral state	0	0	0	0	
T(s=i|s=i,a=a^g^), i drug/aft state	-0.3	*-1.2*	*-1.2*	*-1.2*	From Drug/aft States
T(s=4|s=i,a=a^g^), i drug/aft state	-4	-4	-4	-4	
T(s=i|s=i,a=a^s=*^), i drug/aft state	-0.3	*-1.2*	*-1.2*	*-1.2*	
T(s=4|s=i,a=a^s=*^), i drug/aft state	-4	-4	-4	-4	
T(s=j|s=i,a=a^w^), i!=15 drug/aft state, j next or previous drug/aft state	-0.3	*-1.2*	*-1.2*	*-1.2*	
T(s=4|s=i,a=a^w^), i!=15 drug/aft state	-4	-4	-4	-4	
T(s=14/16|s=15,a=a^w^)	-0.3	*-1.2*	*-1.2*	*-1.2*	
T(s=4|s=15,a=a^w^)	-4	-4	-4	-4	
T(s=j|s=i,a=a^d^), i drug/aft state, j next drug/aft state	-0.3	*-1.2*	*-1.2*	*-1.2*	
T(s=j|s=i,a=a^d^), i drug/aft state, j previous drug/aft state	-0.3	*-1.2*	*-1.2*	*-1.2*	
T(s=4|s=i,a=a^d^), i drug/aft state	-4	-4	-4	-4	
T(s=4|s=1,a=a^g^)	1	1	1	1	Goal
T(s=1|s=1,a=a^s=*^)	0	0	0	0	
T(s=1|s=1,a=a^w^)	0	0	0	0	
T(s=1|s=1,a=a^d^)	0	0	0	0	

Changes during phases in italic.

**Table 3.** Optimal policy across endophenotypes controlled by the RL model (2nd drug phase)

State number	State type	Action	*Q* value
1	Goal	a^g^	2.8967
2	Neutral	a^g^	2.607
3	Neutral	a^s=2^	2.3439
4	Neutral	a^s=3^	2.1074
5	Neutral	a^s=4^	1.8948
6	Neutral	a^s=5^	1.7036
7	Neutral	a^s=6^	1.5317
8	Drug	a^d^	-10.1134
9	Drug-aftereffect	a^d^	-10.3781
10	Drug-aftereffect	a^w^	-10.4882
11	Drug-aftereffect	a^w^	-10.2809
12	Drug-aftereffect	a^w^	-9.7099
13	Drug-aftereffect	a^w^	-8.6469
14	Drug-aftereffect	a^w^	-6.8532
15	Drug-aftereffect	a^w^	-3.9265
16	Drug-aftereffect	a^d^	-5.2928
17	Drug-aftereffect	a^d^	-6.4251
18	Drug-aftereffect	a^d^	-7.3633
19	Drug-aftereffect	a^d^	-8.1408
20	Drug-aftereffect	a^d^	-8.7849
21	Drug-aftereffect	a^d^	-9.318
22	Drug-aftereffect	a^d^	-9.7575

**Table 4.** Agent model parameters across endophenotypes controlled by the RL model

Name	Description	Value
α	MF learning factor	0.05
γ	Discount factor	0.9
d_MB_	MB decay factor	0.01
N_PS_	MB number of updates	50
T_MB_	Temperature for stochastic state update selection	1
ε	Exploration factor	0.1
α_Ctpy_	Cognitive treatment MF learning factor	0.0001, 0.0005, 0.001

**Table 5.** Environment parameters across endophenotypes controlled by the RL model

Name	Description	Value
N_T_	Number of states	22
N_G_	Number goal states	1
N_D_	Number drug/aftereffect states	15
N_n_	Number neutral states	6
N_a_	Number of actions	9
S_0_	Starting state	4
R_p_	Punishment at the end of drug/aftereffect consumption	-4
R_c_	Punishment in drug/aftereffect area	-1.2
R_dd_	Reward at drug consumption (f2,f4)	10
R_dt_	Reward at drug consumption in treatment	-1
R_g_	Reward when entering goal state	1
d_init_	Duration initial (no drug) phase	50
d_drug1_	Duration first drug phase	1000
d_tpy_	Duration treatment phase	1000
d_drug2_	Duration second drug phase	600

## Code accessibility

All models rely on custom code developed in MATLAB (optimized for R2014b) that has been run successfully on multiple OS (iOS, Linux and Windows) on different computers and local servers. The code can be accessed at any time from the repository ModelDB (http://modeldb.yale.edu/239540). The downloadable archive file consists of two folders (respectively, for the neural model and the RL model), which include the entire source code required to replicate the data reported in our Results section. Code available as Extended Data Code File 1.

10.1523/ENEURO.0151-18.2018.ed1 Extended Data Code File 1. To access the source code of both models, visit the ModelDB website (https://senselab.med.yale.edu/modeldb/enterCode.cshtml?model=239540) and download the archive. The source code shows its structure in the commented main files “separate_test.m” and “RunExperimentLearning96.m,” respectively, in the folder “neural_model” and “RL_model.” Download Extended Data C, ZIP file.

## Results

### Simulations from the neural field model

**Figure 3.**
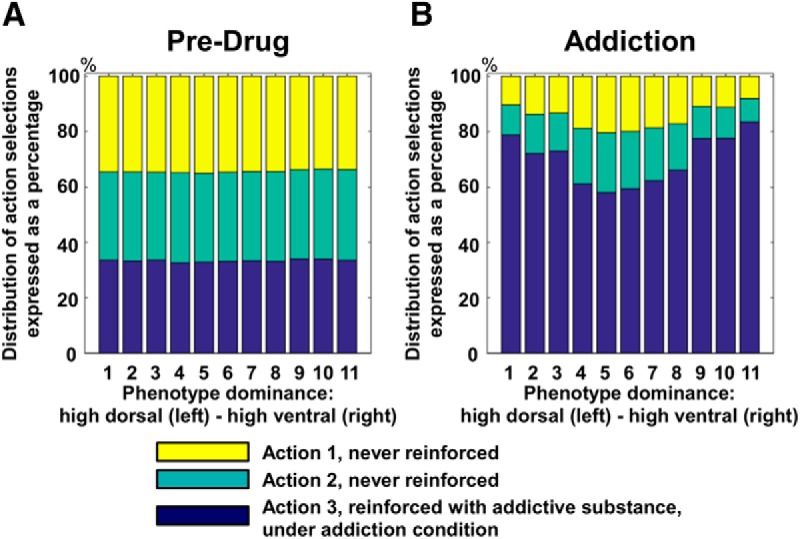
Distribution of action selections across endophenotypes controlled by the neural model. Histograms show how the distribution of simulated action selections changes depending on the endophenotype (11 variations in corticocortical connectivity weights). Thirty random seeds/inputs are used per endophenotype, tested under two stages: pre-drug (***A***) and addiction (***B***). The three colors represent the occurrence of selections of three arbitrary actions. Under the pre-drug stage, no reward is provided, and action selections are triggered by random fluctuation in values of competing sensory inputs. The simulations show the agents adapt to the changes in sensory stimuli and therefore exhibit a near-uniform distribution of action selections. Conversely, under the addiction stage, the action represented in blue is associated with administration of the simulated drug, triggering DA-dependent Hebbian learning in cortico-striatal connectivity, and consequently overselection. Under addiction, the differences among endophenotypes clearly emerge in the selection frequency of the action leading to drug consumption. Asymmetric control (endophenotypes 1–3 and 9–11) leads to a stronger overselection in comparison with balanced control (endophenotypes 4–7), despite identical learning processes and reward encoding.

During all stages, the three stimuli randomly change every few seconds, putatively representing a dynamic fluctuation of values associated with perceived cues in a nonstationary environment. This setup requires the agents to rapidly adapt to these changes, transiently triggering the motor response associated with the most valuable cue, to achieve optimal behavior. During the pre-drug stage, dorsal and ventral circuits perform unbiased selections, collaborating in the generation of a near-optimal sequence of motor selections. All eleven endophenotypes show uniform distributions of action selections, complying with the random distribution of the inputs configurations (Fig. 3*A*). This control stage allows the simulated network to generate transient temporal responses that couple multiple initial states with multiple stable states, in a transient winner-take-all or winner-less competition (Rabinovich et al., 2006; Afraimovich et al., 2008).

During the simulated addiction stage, one of the actions is associated with drug administration (Fig. 3*B*, values represented in blue). Substance use triggers phasic dopamine bursts, leading to Hebbian learning in cortico-striatal connections of both dorsal and ventral circuits (Eq. 3). In recurrent networks, circuit gain increases as a direct function of the weights of reentrant synapses (Amit, 1989). A dopamine response triggered by healthy unexpected rewards would create a bias toward the selection of the reinforced motor response to a perceived cue (Cohen and Frank, 2009; Grahn et al., 2009; Baldassarre et al., 2013). However, drug consumption triggers extra-physiologic dopamine-dependent learning, which in our model results in aberrantly high circuit gain, compromising the ability of all affected circuits to discriminate among different inputs and produce temporal transitions toward multiple stable states (cf. Fiore et al., 2014). The cortico-striatal circuits become overstable and resistant to perturbation caused by a change of input or by noise as they are dominated by parasitic attractors (Hoffman and McGlashan, 2001; Fig. 1*C*). In the ventral cortico-striatal circuit, a parasitic attractor sets and maintains the selection of drug-related goals or outcomes, biasing the action-outcome assessments required for planning. In the dorsal circuit, the same process determines overstable selections of the reinforced motor behavior, generating reactive responses and habits. Importantly, the learning process simulated in our neural model leads to the generation of parasitic attractors in both circuits across all endophenotypes, as all agents eventually reach a fixed threshold in cortico-striatal neural plasticity. Despite the generation of a form of compulsive drug seeking behavior across all endophenotypes, we observe significant differences in motor response patterns as a function of the balance between ventral and dorsal circuits. Specifically, the endophenotypes characterized by unbalanced dorsal or ventral control (i.e., Fig. 3*B*, endophenotypes 1–3 and 9–11) express distributions of motor selections that are significantly more compromised by drug-related aberrant rewards, in comparison with balanced endophenotypes (i.e., Fig. 3*B*, endophenotypes 5–7). The presence of identical learning processes, and the associated attractor formation in both ventral and dorsal circuits, ascribes all phenotypic differences univocally to the only remaining independent variable, which controls corticocortical connectivity and therefore the strength of the biases between circuits. Unbalanced agents are characterized by more frequent drug-related selections as actions leading to drug consumption are selected more frequently than in balanced endophenotypes, in a range between +3% and +45%. This result identifies all phenotypes within the limits of individual differentiation described in the study chosen for behavioral comparison (Gannon et al., 2017).

**Figure 4.**
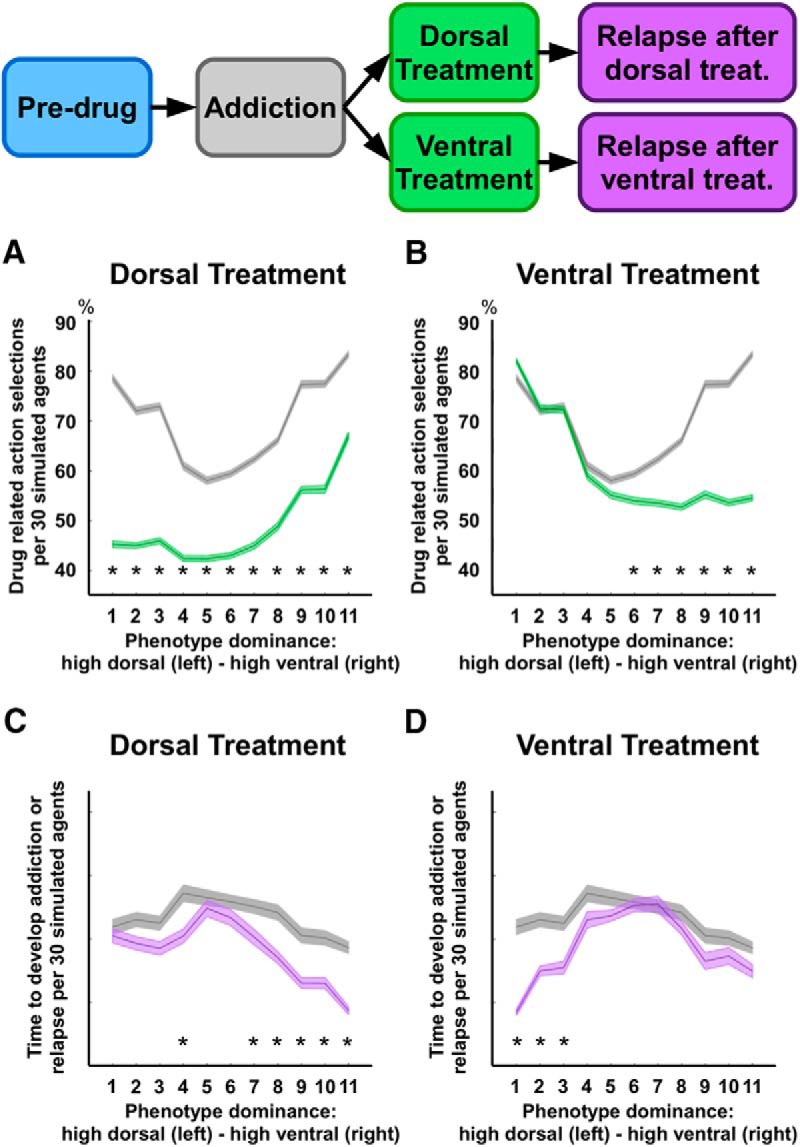
Severity of addiction and relapse time across endophenotypes controlled by the neural model. Shaded error lines report mean and standard error for 30 simulated agents across endophenotypes (11 variations in corticocortical connectivity weights). ***A***, ***B***, Selections of actions leading to substance consumption, as a percentage of the overall number of action selections. In the first case (***A***), we compare the values recorded during the addiction stage with those recorded during the stage of dorsal treatment jointly with abstinence (i.e., drug-related actions do not trigger self-administration of a drug and the treatment targets the dorsal circuit). In the second case (***B***), the comparison involves addiction and ventral treatment (treatment targeting the ventral circuit, during abstinence). ***C***, ***D***, We compare the simulated time required by the 11 endophenotypes to reach an arbitrary threshold of cortico-striatal connectivity during the stage of addiction and during the stage of relapse after either dorsal (***C***) or ventral (***D***) treatment. Within the time of a simulation run, all simulated agents reached the addiction threshold. The two treatments are simulated by restoring either the dorsal/motor (***A–C***) or the ventral/outcome circuit (***B–D***) to the configuration characterizing the pre-drug stage. The percentage of the action selections shows the dorsal treatment is more effective in endophenotypes characterized by high dorsal dominance (***A***), whereas the ventral treatment only has an effect in endophenotypes characterized by high ventral dominance (***B***). Similarly, dorsal and ventral treatments result in long relapse times in endophenotypes characterized by high dorsal and high ventral dominance, respectively; *, significant difference: *p* < .05.

Next, we investigate how the simulated endophenotypes behave during the stages of treatment and relapse. First, we measure the frequency of drug-related action selections during the stages of addiction and treatment (Fig. 4*A*,*B*). Both ventral (goal-oriented) and dorsal (habitual) treatments effectively reduce the number of actions associated with drug consumption, in comparison with baseline addiction. However, the dorsal treatment is more effective for dorsal-dominated endophenotypes and the ventral treatment is more effective for ventral-dominated endophenotypes. These endophenotype-specific treatment effects are further confirmed by our analysis of individual differences under the relapse stage (Fig. 4*C*,*D*): dorsal treatments are more effective in elongating time to relapse for dorsal-dominated endophenotypes, whereas ventral treatments are more successful in delaying relapse for ventral-dominated endophenotypes. This analysis shows that simulated treatments focusing either on the dorsal circuit (and therefore habitual responses) or the ventral circuit (and therefore motivational responses) can have substantially different effects, depending on the balance between dorsal and ventral circuits. Importantly, these differences emerge only after the treatment is applied, where a pre-treatment comparison between compulsive behaviors expressed by the opposite unbalanced endophenotypes (i.e., ventral-dominant or dorsal-dominant) does not show any significant difference in choice selections (Fig. 3*B*, endophenotypes 1–3 and 9–11).

### Simulations from the RL model

**Figure 5.**
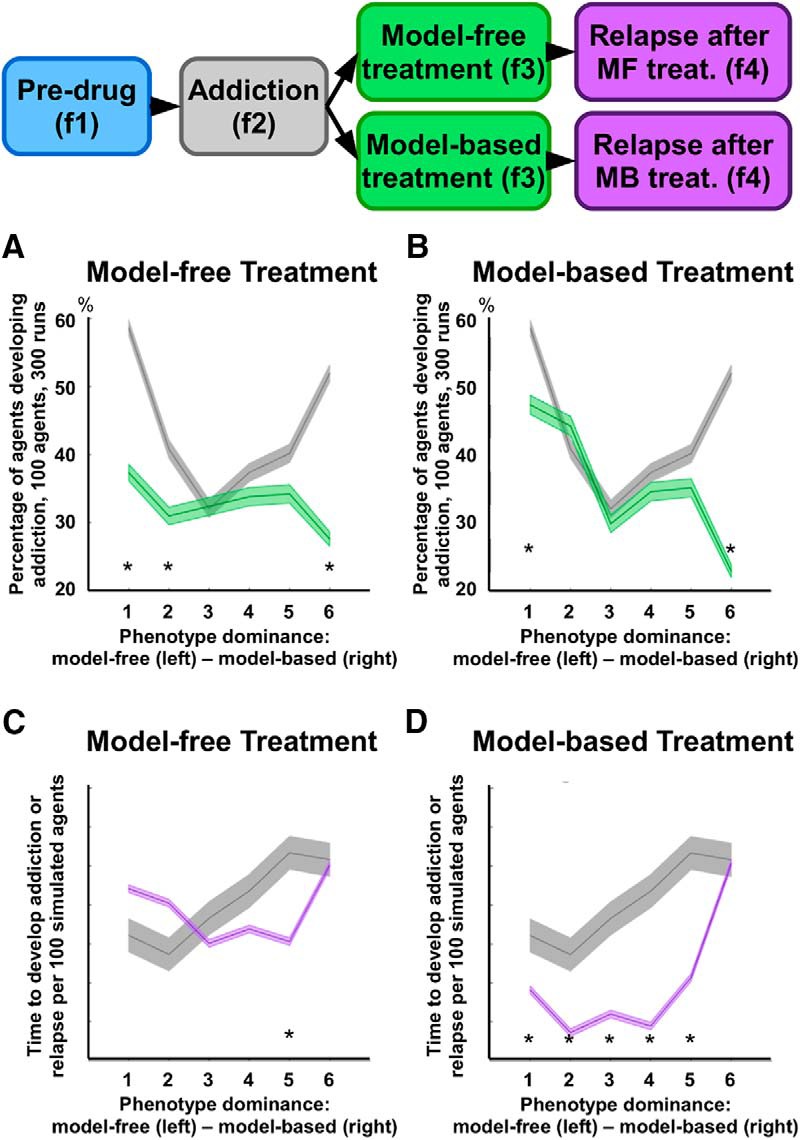
Likelihood to develop addiction and relapse time across endophenotypes controlled by the RL model. Shaded error lines report mean and standard error for ∼100 simulated agents across six endophenotypes (differential balance between model-based and model-free control modalities, *β* = [0, 0.2, 0.4, 0.6, 0.8, 1]). ***A***, ***B***, Percentage of agents developing addiction (i.e., drug-related choices are more frequent than healthy reward-related choices), per endophenotype, under the addiction and treatment phases. In the first case (***A***), the comparison involves data recorded during the phase of addiction and those recorded during the phase of model-free treatment. In the second case (***B***), the comparison involves the phases of addiction and model-based treatment. ***C***, ***D***, Illustration of the simulated time required by the six endophenotypes to reach 95% of action preference toward the drug state, in comparison with action preference recorded during the phase of addiction (f2). In the first case (***C***), the comparison involves the phases of addiction and relapse after model-free treatment, whereas in the second case (***D***), the comparison involves the phases of addiction and relapse after model-based treatment. In terms of action selection ratio, the simulated results show both treatments have a significant effect only on those phenotypes characterized by strong unbalance of control (***A***, ***B***). In terms of relapse, the results show the model-free treatment is on average more successful than the model-based one, as five endophenotypes show no significant difference between the phases of addiction and post-treatment addiction (i.e., the time required to relapse is not significantly different from the time required to develop addiction the first time). Each endophenotype, or parameter selection, was simulated 100 times across the four phases (3050 steps per simulation). Results depend on the statics of the environment, but over similar environments, the results were qualitatively similar; *, significant difference: *p* < .05.

By simulating explicit negative outcomes associated with drug consumption, the RL model allows to measure the likelihood each agent has to develop addiction, as a function of its endophenotype. In our analysis, addiction is defined as a behavior leading to drug selections more frequently than the healthy alternative reward, under the addiction phase. The mean percentage of these addicted agents (over 300 runs) was 43.05%, across endophenotypes, which is consistent with the percentage of rats developing compulsive self-administration of cocaine, as reported in the reference study (∼40% over a period of 5 d; cf. Gannon et al., 2017). Importantly, when considering endophenotype differentiation, the percentage varies significantly: 60.3% for β = 0, 40.3% for β = 0.2, 30.1% for β = 0.4, 36.7% for β = 0.6, 39.3% for β = 0.8, and 51.6% for *β* = 1 (Fig. 5*A*,*B*). This phenotypic differentiation is consistent with well-established data from animal models. For instance, rat strains selectively bred for either high or low voluntary running differ in the likelihood to develop addiction when given free access to cocaine (respectively, ∼35% and ∼60% of each strain develop addiction over a period of 5 d; cf. Smethells et al., 2016). Free access to substances of abuse does not necessarily lead to compulsive behaviors (Piazza et al., 1989; Belin et al., 2011), as addiction varies as a function of factors such as exposure extent, amount of drug delivered, and associated negative effects (Pelloux et al., 2007; Jonkman et al., 2012). Our simulations suggest that endophenotypes with lower chances of addiction are characterized by balanced control modalities. Note that an optimal agent, knowing the environment structure and being able to compute the long-term effects of drug, will never select drug states (Table 3).

Finally, the simulations suggest that the hypothetical treatment targeting model-free control is the most effective, reducing the likelihood to pursue drug-related behaviors for all endophenotypes (Fig. 5*A*). In contrast, the model-based treatment appears to be less effective for all endophenotypes, with the exception of the purely model-based one (β = 1; Fig. 5*B*). Under the relapse phase, our data confirm that the simulated treatments significantly differ in their effectiveness across the proposed endophenotypes, also suggesting the treatment targeting model-free control is the most successful in prolonging relapse time (Fig. 5*C*,*D*). Relapse time after model-free treatment is mostly similar to the time required to develop addiction behavior before any treatment (Fig. 5*C*). At the opposite side of the control spectrum, the model-based treatment shows a positive effect only for the purely model-based endophenotype. All remaining endophenotypes show relapse times significantly shorter than those recorded for the first development of addiction (β= 1; Fig. 5*D*).

## Discussion

Individual differences in stress and anxiety responses (Dilleen et al., 2012; Jimenez and Grant, 2017), social dominance (Morgan et al., 2002; Covington and Miczek, 2005), aggressive temperament (McClintick and Grant, 2016), preference for saccharine (Carroll et al., 2002), sensation or novelty seeking (Suto et al., 2001; Nadal et al., 2002; Belin et al., 2011; Flagel et al., 2014), impulsivity (Perry and Carroll, 2008; Verdejo-García et al., 2008; Dalley et al., 2011), and sensitivity to rewards (Belcher et al., 2014) have all been found in both animal models and clinical studies in humans to be associated with addiction vulnerabilities, and in particular with the likelihood to develop and maintain addiction, or to resist to treatment (Piazza et al., 1989; Belin et al., 2016; Everitt and Robbins, 2016). However, investigations into the mechanisms underlying this phenotypic differentiation in addiction has so far revealed few neural or computational candidates, which are found to be associated with diverse and dissociable behavioral traits. An important example is represented by the endophenotypic differentiation reported in the expression and reactivity of striatal D2 dopaminergic receptors, which is found to be negatively correlated with the traits of impulsivity (Dalley et al., 2007), social dominance (Morgan et al., 2002), and sensitivity to rewards (Belcher et al., 2014) and nonlinearly correlated with novelty preference (Flagel et al., 2014). The overlap of this endophenotypic trait across multiple, noncoexisting, phenotypes associated with addiction vulnerabilities suggests other neural or computational mechanisms have yet to be identified to allow accounting for the reported variety in behavioral traits.

Here, we have presented a neural field model, augmented by an RL model, to expand on existing neuropsychological and computational accounts of addiction. Our models propose a theoretical investigation into the interaction among cortico-striatal circuits or behavioral control modalities, and the effects this interaction has on addiction development and treatment response. As described in classic models (Redish, 2004, 2008; Dayan, 2009), we have assumed that overevaluation of a drug leads to aberrant dopamine release and associated overlearning in multiple DA targets (Volkow and Morales, 2015; Koob and Volkow, 2016). In the neural field model, this mechanism results in the dysregulation of the circuit gain and associated dynamics of both ventral and dorsal cortico-striatal circuits (Fiore et al., 2014; Hauser et al., 2016). In the integrated model-based and model-free RL model, sequential choice behavior is confounded by the presence of a high immediate reward (drug state). This leads to misrepresent the negative outcomes following drug consumption, if their distribution across states and time is sufficiently complex to escape the capabilities of the agent to correctly represent the environment (Doll and Daw, 2016; Sadacca et al., 2016). We found that both models jointly indicate that the balance between neural circuits or behavioral control modalities is a candidate neurocomputational mechanism characterizing endophenotypes in addiction. The neural and RL models converge in suggesting that individuals characterized by balanced behavioral control between reward-seeking or planning (ventral circuit/model-based) and reactive or habitual responses (dorsal circuit/model-free) would have a reduced chance to develop addiction and decreased severity of symptoms if developing addiction. We propose that this neurocomputational mechanism may be interacting with other known endophenotypic differentiations, such as alterations of D2 receptors in the striatum (Morgan et al., 2002; Nader and Czoty, 2005; Dalley et al., 2007; Volkow et al., 2007; Belcher et al., 2014; Flagel et al., 2014) or differences in learning rates (Gutkin et al., 2006; Piray et al., 2010), to generate the multifaceted behavioral traits that have been reported in literature to be associated with addiction vulnerabilities.

In our neural model, ventral and dorsal circuits are mostly in phase in their selections under the pre-drug stage, exhibiting synchronous transient stability of neural activity and enhancing the overall ability of the system to adapt to changing stimuli (i.e., the two circuits adapt to the input changes with a similar pace and synchronize in their selection). Under the addiction stage, the two circuits are mostly pulled toward the parasitic attractor state associated with drug consumption, and they occasionally select the competing non-drug stimuli. If only one of the two systems performs a selection outside of the attractor, the difference in selection generates a dissonance or interference. In neural endophenotypes characterized by unbalanced control, this dissonance is solved by one circuit taking the lead, so that both systems eventually converge on the selection of the dominant circuit. These dynamics result in limited opportunities to generate non-drug-related responses to the external stimuli, as they can only be generated by the dominant circuit. Conversely, in balanced control endophenotypes, if any of the two circuits ignores the drug-stimulus and selects a competing option, the resulting dissonance can trigger a state transition pulling out the parasitic attractor states associated with substance use. The endophenotypes in our simulations vary only in the parameters regulating the balance between circuits, as dopamine-driven learning processes established between cortex and striatum (Eq. 3) do not vary across endophenotypes, resulting in identical habit formation and drug-related biases in the outcome representations. Thus, our proposed phenotypic differentiation does not interfere with the usual role ascribed to the ventral and dorsal circuits as, respectively, implicated in the initial reward-seeking phase in addiction (Belin and Everitt, 2008; Willuhn et al., 2012) and the subsequent consolidation of stimulus-response, habitual, association (Everitt and Robbins, 2013, 2016). However, our simulated dynamics show that, after addiction is developed, systemic overstability can be reduced or further enhanced, depending on the corticocortical biases between cortico-striatal circuits. In turn, this modulation of system stability can foster or further impair input discrimination and motor response versatility, affecting addiction symptomatology. As a result, our neural model shows phenotypic variability emerging after the presentation of the reward simulating the drug and addiction is developed, in a gradient of overselection of drug-related actions.

With the RL model, we investigate whether the balance between model-based and model-free modalities would also increase the robustness of the system against the selection of drug states in a more complex environment and in presence of explicit negative outcomes. Similar to the neural model, a system with balanced control modalities introduces more diversity in action selection during exploration, reducing (yet not cancelling) the chances of developing maladaptive reactive responses. This increased diversity and overall reliability are likely to be induced by a higher redundancy and diversification of the system. While both components may fail, the causes of failures are not necessarily correlated. The model-based system can fail due to its sensitivity to cognitive resources but it is more efficient in encoding previous experience of the agent. On the other hand, the model-free component is affected by limited exploration but it is reliable in its selections, which are not affected by the availability of cognitive resources. Consistent with the neural model, differentiations in behaviors among endophenotypes emerge in an inverted-U shape, where unbalanced control system are the most vulnerable to developing addiction.

The phenomenon of relapse is more elusive and the two models do not fully converge on this aspect. To investigate this phenomenon, we have adapted the complexity of real world treatments to the capabilities of our simulated agents and environments, where we can easily manipulate or extinguish consolidated memory, but we cannot engage all other aspects commonly involved in addiction treatment, such as cognitive or emotional functions or developing new behavioral strategies to compete with drug-related habits. Therefore, we implemented two compartmentalized treatments that we consider as ideal reference models that target only a single decision system or circuit. These putatively represent treatments capable of affecting only drug-related emotional/value or habitual/motor associations. In the neural model, balanced dorsal and ventral endophenotypes respond well to both types of simulated treatments. For the unbalanced endophenotypes, however, only the appropriate treatment, targeting the dominant neural circuit, is effective. The simulations in the RL model do not show the same symmetric effects for the two treatments: the model-free treatment is effective for most endophenotypes, whereas the model-based treatment is mostly unsuccessful, with short relapse times across all endophenotypes, but the purely model-based one. The latter result is possibly due to the learning process characterizing the model-based component, which is affected by conflicting information as drug use is associated with both positive and negative outcomes, experienced by the agent when entering the drug state under different phases.

It is worth noting that habitual and goal-oriented behaviors have neural representations in the dorsal and ventral cortico-striatal circuits, respectively, but they do not fully overlap with model-based and model-free control modalities in RL (Dolan and Dayan, 2013). Nonetheless, the neural and RL models independently simulate choices among competing options in addiction. Thus, we have been able to test our hypothesis of endophenotypic differentiation under two complementary levels in Marr’s tri-level of analysis: the neural implementation and the algorithmic level (Marr and Poggio, 1976). This multilevel modeling approach has been often used in computational psychiatry (Maia and Frank, 2011; Montague et al., 2012; Adams et al., 2016; Hauser et al., 2016; Huys et al., 2016) to highlight model convergence and associate specific neural structure and dynamics with mathematical formalizations of optimal and suboptimal behavior in RL. The convergence of neural and RL models on important predictions also provides more confidence in the reliability of the identified computational mechanisms underlying addiction and the associated characterization of endophenotypes. Specifically, both models indicate individuals with unbalanced cortico-striatal activity or control modality are at higher risk of developing addiction and relapse after any treatment. Thus, independent of phenotypic-specific treatments, our results suggest that individuals with these traits would require a prolonged or more intense treatment, in comparison with balanced endophenotypes. Finally, when considering phenomena that are divergent across both models (e.g., response across endophenotypes to our simulated treatments), our findings still demonstrate that important endophenotypic features might remain undetected in terms of pre-treatment observable behavior. The models showed that opposite unbalanced agents resulted in similar addictive behaviors and vulnerabilities, but diverged in treatment response, potentially informing the development of precision interventions. Further studies will be required to provide empirical validation of our models. For example, computational analysis of fMRI data can be used to test effective connectivity among cortico-striatal circuits (Friston et al., 2003), in conjunction with cognitive tasks targeting the model-based and model-free control systems.

## Synthesis

Reviewing Editor: Gustavo Deco, Universitat Pompeu Fabra Decisions are customarily a result of the Reviewing Editor and the peer reviewers coming together and discussing their recommendations until a consensus is reached. When revisions are invited, a fact-based synthesis statement explaining their decision and outlining what is needed to prepare a revision will be listed below. The following reviewer(s) agreed to reveal their identity: Marc Exton-McGuinness, Vincent Van Waes.

Rev 1:

The authors describe a computational model for the propensity of different striatal-based endophenotypes to drug addiction and simulate the effect of ventral/dorsal based treatments on relapse. The simulations are well conducted using established computational frameworks. The rationale is strong, as there is significant heterogeneity in addiction; moreover, endophenotypic differences may predict optimal treatment routes. My main criticism is the foundation of the dorsal/ventral (model-free/based) endophenotype model is not communicated as clearly as it could be in the introduction and methods; however, my points are all relatively minor and the study would be of interest to a broad readership, suitable for publication with minor modification. A point by point review is provided below.

1) Line 39: “two fundamental alterations...” It would be useful to explicitly state at some point in the introduction the hypothesis of dorsal/ventral balance in addiction, as this forms the basis of the model construction.

2) Line 115-117: “neural mass model that has been validated”. It would be helpful to the reader to explicitly cite here to point the reader in the direction of the validation study(s).

3) Line 136: “construct validation...” I'm unsure they truly provide for validation of each other, as this is only true if they predict the same outcome - which they don't necessarily?

4) Line 195: “these putative endophenotypes...” it is not immediately clear at this point in the manuscript what endophenotypes are being tested (relates to point 1).

5) Line 204: “simulated reverting learning process in either dorsal of ventral...” I think it would be useful to link this to existing addiction treatment programs? What current behavioural treatments/counselling methods would these simulated treatments map onto?

6) Also, it might be useful to have a ‘no treatment’ control simulation to compare time to relapse following an abstinence period equivalent to the treatment period, if this can be captured by the model?

7) Line 402: It would be useful here to critically appraise the reference study (Gannon et al. 2017) definition of compulsive self-administration (i.e. time-out responding) contrasted with the present study (selection of drug over alternatives), as these are not the same.

8) Line 525: There is an opportunity here for some interesting discussion related to the hypothesis that addiction is driven by a maladaptive habit (Everitt and Robbins, 2005). Perhaps the authors could comment here on whether they believe it is most useful to conceptualise addition as a ‘model-free’ habit for the purposes of treatment given their finding the model-free treatment was most effective generally; vs the incentive-salience hypothesis (Robinson and Berridge 2008)?

Rev 2:

This manuscript entitled “A Multilevel Computational Characterization of Endophenotypes in Addiction” is very well written and fascinating. The authors used two models (a neural model based on cortico-striatal circuit dynamics, and an algorithmic model of reinforcement learning) in an attempt to better understand the origin of interindividual differences in the vulnerability to develop an addiction. This is an elegant, novel and complementary approach to address this important theoretical question. Overall, this paper is very good, although quite difficult to read for a neophyte in computational neuroscience. It is important to note that the authors have also a good knowledge of the neurobiological mechanisms underlying addiction-related behavior. For these reasons, I think the manuscript deserve to be published in eNeuro.

## Author Response

Dear editor and reviewers,

Thank you for considering our manuscript and for highlighting the merits of our study. We have now revised our manuscript after carefully considering and addressing all your previous concerns. We hope you will find this revised version appropriate for publication in eNeuro. Below, we provide a point-by-point reply to the reviewers’ comments. Please note our inline responses and excerpts from the revised manuscript.

Reviewer #1:

The authors describe a computational model for the propensity of different striatal-based endophenotypes to drug addiction and simulate the effect of ventral/dorsal based treatments on relapse. The simulations are well conducted using established computational frameworks. The rationale is strong, as there is significant heterogeneity in addiction; moreover, endophenotypic differences may predict optimal treatment routes. My main criticism is the foundation of the dorsal/ventral (model-free/based) endophenotype model is not communicated as clearly as it could be in the introduction and methods; however, my points are all relatively minor and the study would be of interest to a broad readership, suitable for publication with minor modification. A point by point review is provided below.

1) Line 39: “two fundamental alterations...” It would be useful to explicitly state at some point in the introduction the hypothesis of dorsal/ventral balance in addiction, as this forms the basis of the model construction.

Response 1: Thank you for your encouraging and constructive comments. We fully agree that further clarifying the theoretical foundation of ventral/dorsal circuits could help the reader better understand our study. We have addressed this concern by modifying the text in the introduction as follows (line 62):

“Here we propose a theoretical investigation into the interaction between ventral and dorsal cortico-striatal circuits and the associated behavioral control modalities. Several studies have emphasized that addiction is associated with alterations of ventral and dorsal cortico-striatal circuits, and of motivations and habits (Volkow and Morales, 2015; Everitt and Robbins, 2016; Koob and Volkow, 2016). However, the role played by the interaction between the two neural circuits or between the two behavioral control modalities in generating intersubject variability in addiction, has been so far neglected. To investigate this interaction, we use two models to simulate neural dynamics and algorithmic (or normative) choice selections in a multiple-choice task involving drug and non-drug rewards. Then we test these models under different conditions of circuit or control modality dominance (i.e. simulated endophenotypes).”

At the beginning of the Methods section we have further amended the text, as follows (line 104):

“In brief, we present two complementary models simulating endophenotypic differences and their effects on addiction development and treatment response. In the models, intersubject differences are expressed in terms of either neural circuit dominance (i.e. ventral or dorsal circuit) or control modality dominance (i.e. model-based or model-free) in determining behavioral selections.”

2) Line 115-117: “neural mass model that has been validated”. It would be helpful to the reader to explicitly cite here to point the reader in the direction of the validation study(s).

Response 2: We have now modified the main text to include references providing validation for both the neural model and the RL model (line 134):

“The two models comprise a neural mass model that has been validated and described in the context of choice behavior and dopaminergic modulation (Fiore et al., 2016; Hauser et al., 2016; Fiore et al., 2018) and a normative or algorithmic model based upon standard RL schemes (Sutton and Barto, 1998).”

3) Line 136: ‘construct validation...’ I'm unsure they truly provide for validation of each other, as this is only true if they predict the same outcome - which they don't necessarily?

Response 3: We fully agree that there are important differences in some of the predictions formulated by the two models (e.g. concerning treatment response), as well as significant convergence on other important predictions (e.g. phenotypic robustness in the face of addictive rewards for “balanced” endophenotypes). We have now rephrased this sentence to emphasize that the validation is limited to the converging predictions (line 157):

“The unique aspect of this complementary modeling approach is that converging results from neural and algorithmic models can validate each other, as process and implementation theories (i.e., synaptic and dynamical mechanisms) complement the normative principles formalized in the RL model.”

4) Line 195: ‘these putative endophenotypes...’ it is not immediately clear at this point in the manuscript what endophenotypes are being tested (relates to point 1).

Response 4: As proposed in the above response to comment #1, we have modified both the Introduction and the Methods section to provide more explicit details about endophenotypic differentiation. We report these changes here for your convenience. In the introduction (line 63):

“Here we propose a theoretical investigation into the interaction between ventral and dorsal cortico-striatal circuits and the associated behavioral control modalities. Several studies have emphasized that addiction is associated with alterations of ventral and dorsal cortico-striatal circuits, and of motivations and habits (Volkow and Morales, 2015; Everitt and Robbins, 2016; Koob and Volkow, 2016). However, the role played by the interaction between the two neural circuits or between the two behavioral control modalities in generating intersubject variability in addiction, has been so far neglected. To investigate this interaction, we use two models to simulate neural dynamics and algorithmic (or normative) choice selections in a multiple-choice task involving drug and non-drug rewards. Then we test these models under different conditions of circuit or control modality dominance (i.e. simulated endophenotypes).”

At the beginning of the Methods section, as follows (line 104):

“In brief, we present two complementary models simulating endophenotypic differences and their effects on addiction development and treatment response. In the models, intersubject differences are expressed in terms of either neural circuit dominance (i.e. ventral or dorsal circuit) or control modality dominance (i.e. model-based or model-free) in determining behavioral selections.”

5) Line 204: “simulated reverting learning process in either dorsal of ventral...” I think it would be useful to link this to existing addiction treatment programs? What current behavioural treatments/counselling methods would these simulated treatments map onto?

Response 5: Thank you for this suggestion. We fully agree that it would be very informative to both clinician and non-clinician readers, if we can establish a clear parallel between addiction treatment currently carried out in clinics and our simulated ones. Such comparison would also directly support the goal of computational psychiatry (i.e. bridging the gap between basic research and clinical work). Our “stylized therapeutic interventions” have been conceived with a twofold purpose as we aim to 1) report neural dynamics and information processing expressed by the different endophenotypes under significantly different conditions, and 2) represent as much as possible realistic behavioural and cognitive treatments. Furthermore, the complexity of real treatments had to be scaled and adapted to the capabilities of our simulated agents and environments. Our simulations easily allow to manipulate or extinguish consolidated memory, but they cannot engage (at least not in this initial investigation) other cognitive or emotional functions that are commonly involved in addiction treatment. Furthermore the environment does not allow the formation of newly learned behaviors to avoid substance use. As a result, we implemented two compartmentalised treatments that we consider as ideal or abstract reference models that artificially target only a single decision system or circuit to emphasize the different results on the simulated endophenotypes. Despite these limitations, we believe a parallel between our two simulated treatments and actual behavioural and cognitive treatments can provide important insight into plausible processes and neural substrates underlying differences in addiction vulnerabilities and treatment responses.

The text has been modified in the Methods as follows (line 226):

“The treatments are simulated by reverting the learning process in either the dorsal or the ventral cortico-striatal circuit, respectively representing an intervention that would block or extinguish either the habitual drug-related response (an ideal behavioral treatment) or the drug-related emotional and value association (an ideal cognitive treatment).”

In the Discussion we now report (line 546):

“The phenomenon of relapse is more elusive and the two models do not fully converge on this aspect. To investigate this phenomenon, we have adapted the complexity of real world treatments to the capabilities of our simulated agents and environments, where we can easily manipulate or extinguish consolidated memory, but we cannot engage all other aspects commonly involved in addiction treatment, such as cognitive or emotional functions or developing new behavioral strategies to compete with drug-related habits. Therefore, we implemented two compartmentalized treatments that we consider as ideal reference models that target only a single decision system or circuit. These putatively represent treatments capable of affecting only drug-related emotional/value or habitual/motor associations.”

6) Also, it might be useful to have a ‘no treatment’ control simulation to compare time to relapse following an abstinence period equivalent to the treatment period, if this can be captured by the model?

Response 6: Thank you for this helpful suggestion. A “no treatment” control simulation may imply two scenarios. The first scenario involves agents that are not affected by any manipulation and are simply tested as they are at the end of the addiction phase, in a new run under the no-drug condition. In this case, however, time of relapse would be equal to zero across all endophenotypes, as for the neural model we consider agents have relapsed if they reach the neural configuration recorded under the addiction phase. The second scenario entails a memory decay, assuming that simulated addict individuals kept away from both the stimuli eliciting drug-related behaviour and the substance of abuse itself, without any other treatment, would partially “forget” drug-related motivations and actions, due to a diminished strength of drug-related motor and goal-oriented connectivity. We have tested this second scenario under different levels of decay or “forgetting rate”. As exemplified in the results we report here, we found that the time of simulated relapse was a fraction of the time required to develop addiction the first time, as stronger decays resulted in longer time in a stable proportion across endophenotypes. Given these results, we have not amended the manuscript to describe this test, as we deemed it to be not informative of a real abstinence without treatment scenario.

7) Line 402: It would be useful here to critically appraise the reference study (Gannon et al. 2017) definition of compulsive self-administration (i.e. time-out responding) contrasted with the present study (selection of drug over alternatives), as these are not the same.

Response 7: We appreciate your concern regarding this matter. There are indeed important differences between task used for our two models and the standard operant conditioning chambers used in the chosen reference study by Gannon et al. (2017). This is not an uncommon choice: despite the fact “time-out” procedures are very common in animal models of addiction, artificial agents mostly rely on multiple-options simulated set-ups (see e.g. Redish, 2004 and subsequent). This choice of a richer environment allows to establish a better approximation of both real world and experimental conditions, as animals are always confronted with different types of stimuli and rewards, even in the simplified environment of an operant chamber (e.g. a rodent might be driven by the desire to explore the chamber). We have modified the text at the beginning of the Methods section as follows, to better describe our reasoning on this matter (line 120):

“Importantly, the task setup chosen for both of our proposed models involves the selection of a drug reward over explicit non-drug related alternatives; in contrast, the chosen empirical study utilizes a time-out responding paradigm, where the only explicit non-drug related behavior (a lever-press) is not rewarded. As for most studies simulating addiction (e.g. see: Redish, 2004), we believe the choice to present our simulated agents with a richer set of options (i.e. more than one) does not invalidate a parallel between simulated and real data. We consider the simulated competing options as a proxy for the many conflicting stimuli and associated behaviors that animals have access to, even in the limited environment of a standard operant conditioning chamber. Thus, our focus is on perturbing the balance between the dorsal/model-free and the ventral/model-based systems, to compare our simulated behavioral differentiations in the escalation and compulsive selection of drug-related actions with the data reported in the chosen laboratory study.”

8) Line 525: There is an opportunity here for some interesting discussion related to the hypothesis that addiction is driven by a maladaptive habit (Everitt and Robbins, 2005). Perhaps the authors could comment here on whether they believe it is most useful to conceptualise addition as a ‘model-free’ habit for the purposes of treatment given their

finding the model-free treatment was most effective generally; vs the incentive-salience hypothesis (Robinson and Berridge 2008)?

Response 8: Thank you for suggesting this possible interpretation of our data. It is indeed interesting that the MF treatment in the RL model is the most effective across endophenotypes, including the purely model-based one. However, the two models do not converge on this prediction, as the neural model shows a significant differentiation in predicted outcomes for the two treatments, depending on circuit dominance. Indeed, the dorsal treatment in the neural model, which represents the intervention in the neural substrate mainly responsible for MF control, fails to have a significant effect for those phenotypes unbalanced in favour of the ventral circuit, which respond well to the ventral treatment (loosely equivalent to MB treatment). This divergence is due to the different constructs characterising the two models as well as the different key assumptions at the basis of the respective idealised treatments. We have amended the text to clarify merits and weaknesses underlying this difference at the end of the Discussion section, as follows (line 575):

“The convergence of neural and RL models on important predictions also provides more confidence in the reliability of the identified computational mechanisms underlying addiction and the associated characterization of endophenotypes. Specifically, both models indicate individuals with unbalanced cortico-striatal activity or control modality are at higher risk of developing addiction and relapse after any treatment. Thus, independent of phenotypic-specific treatments, our results suggest that individuals with these traits would require a prolonged or more intense treatment, in comparison with balanced endophenotypes. Finally, when considering phenomena that are divergent across both models (e.g. response across endophenotypes to our simulated treatments), our findings still demonstrate that important endophenotypic features might remain undetected in terms of pre-treatment observable behavior. The models showed that opposite unbalanced agents resulted in similar addictive behaviors and vulnerabilities, but diverged in treatment response, potentially informing the development of precision interventions.”

Reviewer #2:

This manuscript entitled “A Multilevel Computational Characterization of Endophenotypes in Addiction” is very well written and fascinating. The authors used two models (a neural model based on cortico-striatal circuit dynamics, and an algorithmic model of reinforcement learning) in an attempt to better understand the origin of interindividual differences in the vulnerability to develop an addiction. This is an elegant, novel and complementary approach to address this important theoretical question. Overall, this paper is very good, although quite difficult to read for a neophyte in computational neuroscience. It is important to note that the authors have also a good knowledge of the neurobiological mechanisms underlying addiction-related behavior. For these reasons, I think the manuscript deserve to be published in eNeuro.

Response 1: We thank the reviewer for this very positive and encouraging review and for the appreciation expressed for our efforts in carrying out this study.
